# A method for the fast and photon‐efficient analysis of time‐domain fluorescence lifetime image data over large dynamic ranges

**DOI:** 10.1111/jmi.13128

**Published:** 2022-06-23

**Authors:** Romain F. Laine, Chetan Poudel, Clemens F. Kaminski

**Affiliations:** ^1^ Laser Analytics Group, Department of Chemical Engineering and Biotechnology University of Cambridge Philippa Fawcett Drive Cambridge UK; ^2^ Medical Research Council Laboratory for Molecular Cell Biology (LMCB) University College London Gower Street London WC1E 6BT; ^3^ Department of Chemistry University of Washington Seattle, WA 98195 USA

**Keywords:** center of mass, Fiji plugin, FLIM, quantitative analysis

## Abstract

Fluorescence lifetime imaging (FLIM) allows the quantification of sub‐cellular processes in situ, in living cells. A number of approaches have been developed to extract the lifetime from time‐domain FLIM data, but they are often limited in terms of speed, photon efficiency, precision or the dynamic range of lifetimes they can measure. Here, we focus on one of the best performing methods in the field, the centre‐of‐mass method (CMM), that conveys advantages in terms of speed and photon efficiency over others. In this paper, however, we identify a loss of photon efficiency of CMM for short lifetimes when background noise is present. We subsequently present a new development and generalization of CMM that provides for the rapid and accurate extraction of fluorescence lifetime over a large lifetime dynamic range. We provide software tools to simulate, validate and analyse FLIM data sets and compare the performance of our approach against the standard CMM and the commonly employed least‐square minimization (LSM) methods. Our method features a better photon efficiency than standard CMM and LSM and is robust in the presence of background noise. The algorithm is applicable to any time‐domain FLIM data set.

## 1 INTRODUCTION

Fluorescence lifetime imaging (FLIM) provides a functional read‐out of phenomena occurring at the molecular level and, in contrast to intensity‐based imaging, informs not only on the location of a fluorescent label but also its local environment. It is now widely used in biological research to quantify a plethora of cellular parameters, including ion concentrations, temperature or viscosity.^1^, ^2^, ^3^, ^4^ FLIM has been implemented in numerous modalities, which include point‐scanning and wide‐field imaging methods^5^ both in the time‐ and frequency‐domains.^6^ In time‐domain approaches, the lifetime is commonly extracted from FLIM data using non‐linear least‐square minimization (LSM), for which a number of open‐source packages are available.^7^, ^8^ Typically, LSM requires the acquisition of many temporal gates to yield accurate measurement of the lifetime, and this leads to long acquisition times. While some methods such as rapid lifetime determination (RLD) allow video rate FLIM,^9^ they are precise only over a comparatively small range of lifetimes.^10^ With recent technological advancement, in particular in the complementary metal‐oxide‐semiconductor (CMOS) and single‐photon avalanche diode (SPAD) technologies, FLIM has gained a significant improvement in temporal resolution without loss in lifetime precision,^11^, ^12^ essentially through efficient parallelization in order to circumvent pile‐up effects.^13^ These developments have renewed the interest in fast computational strategies and algorithms for lifetime determination. Some examples include the analogue mean‐delay method for fast and precise lifetime estimation limited to single‐exponential decays,^14^, ^15^ Bayesian analysis methods which offer distinct advantages when photon counts are low^16^, ^17^ and global analysis methods that estimate the global fluorescence lifetime in an image by summing photons across all pixels at the cost of losing spatial information of lifetime distributions.^8^, ^18^ One of these fast computational methods is the so‐called centre‐of‐mass method (CMM), in which the first moment (centre‐of‐mass) of the fluorescence decay is used as a lifetime estimator.^19^ The CMM algorithm is non‐iterative, computationally efficient and has been shown to be orders of magnitude faster when compared against standard LSM techniques.^20^ Owing to this faster speed, it has been implemented on‐chip, for on‐the‐fly lifetime estimation.^21^, ^22^

In CMM, a temporal window (the analysis window) within the acquired decay time‐range (the acquisition window) is chosen to compute the centre‐of‐mass. We recall that CMM is based on the calculation of the first moment (centre‐of‐mass, CM) of a fluorescence decay:

(1) $$CM=\frac{\int_{0}^{T}I\left(t\right)tdt}{\int_{0}^{T}I\left(t\right)dt}=\tau -\frac{Te^{-\frac{T}{\tau }}}{1-e^{-\frac{T}{\tau }}},$$

where $I\left(t\right)=I_{0}e^{-\frac{t}{\tau }}$ is the fluorescence decay, τ is the fluorescence lifetime and *T* the measurement window over which the signal is measured. For T≫τ, CM∼τ as the second term vanishes, but for short measurement windows, a correction needs to be applied to infer τ and take into account the finite size of the measurement window. This can be achieved using either an iterative approach or a look‐up table.^21^

In this paper, we identify that when background noise is present, lifetimes that are short with respect to the size of the analysis window are evaluated inefficiently by the standard CMM leading to an imprecise estimation of the lifetime. Exploiting the flexibility in the choice of analysis window, we present a generalization of CMM called F3‐CMM to correct for this effect by applying the fusion of three lifetime images obtained with adapted temporal analysis windows. Our approach extends the dynamic range of fluorescence lifetimes that can be analysed with CMM at high photon efficiency. The method potentially works for all time‐domain FLIM data sets and could also be implemented on‐chip to achieve fast and real‐time FLIM analysis (millisecond timescales for standard 256×256×256 FLIM data), similar to standard CMM.^19^, ^21^

## 2 RESULTS

### 2.1 Photon efficiency of CMM and LSM lifetime estimations

To compare the performance of CMM with other commonly used analysis methods, we modelled time‐correlated single photon counting (TCSPC) data with Monte‐Carlo simulations of photon arrival times (see Figure S1 and Materials and Methods for details). The software is distributed with this work and includes a Gaussian model of the instrument response function (IRF), photon noise, the effects of after‐pulsing (Ap)^23^ and laser repetition rate. The Ap represents the fraction of photons in the background compared to those in the decay and is here a metric of the amount of noise on the background level. The software tool allows the simulation of realistic FLIM data that can be directly imported into our analysis software described further below, or into the commonly used FLIMfit software.^8^ In a previous error analysis of CMM,^19^ simulations did not include the effect of background noise on algorithm performance, although background correction is an essential step in CMM.^22^ Here, we use simulations for typical acquisition conditions for TCSPC (time window T=25 ns, 256 time bins, a Gaussian IRF centred at 3.2 ns with a standard deviation of 150 ps and containing a total of 108 photons) to estimate the quality of the lifetime estimation of F3‐CMM and compare it to the common LSM method. A useful parameter to estimate the photon efficiency of a lifetime method is the *F*‐value^24^ which compares the signal‐to‐noise ratio (SNR) for a photon counting method to the precision of the measured lifetime:

(2) $$F=\sqrt{N}\frac{\sigma_{\tau }}{\tau },$$

where *N* is the total number of photons, τ is the fluorescence lifetime and $\sigma_{\tau }$ is the standard deviation of the lifetime measurement obtained from repeated measurements. For an ideal method, the photon efficiency reaches the minimum value of F=1, for other cases F>1.

**FIGURE 1:**
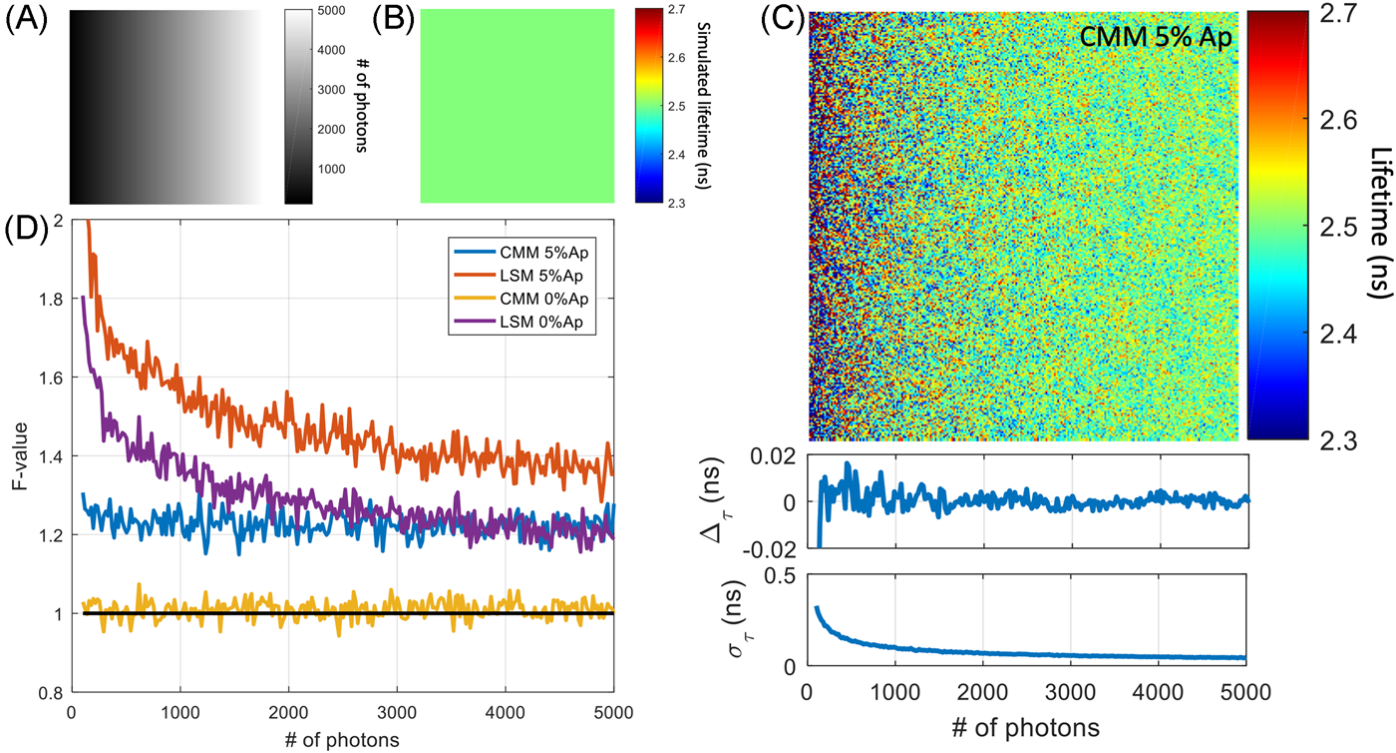
Effect of photon counts on lifetime determination in presence and absence of background noise. (A) Image of the total photon counts showing the gradient of photons from left (100 photons) to right (5000 photons). (B) Uniform simulated lifetime image (2.5 ns). (C) Top: Lifetime image obtained from CMM analysis with 5% after‐pulsing. Middle: Deviation of measured lifetime from the simulated lifetime (Δτ) as a function of the number of photons (from 1024 repeats). Bottom: Standard deviation of the measured lifetime ($\sigma_{\tau }$) as a function of the number of photons (from 1024 repeats). (D) *F*‐value as a function of the number of photons (from 1024 repeats) for CMM and LSM in presence (5% Ap) and absence of background (0% Ap). When 5% after‐pulsing is applied, the background is corrected by removing the average of the first ∼20 times gates for CMM and by background level fitting for LSM. Ap: after‐pulsing. The analysis window used the full acquisition window: 0–25 ns

Figure 1 shows fluorescence lifetime images from simulations of a sample with uniform lifetime (2.5 ns). The simulation features a gradient of photon counts (from 100 to 5000 photons) with background noise levels of Ap = 0% and Ap = 5% and was analysed with both standard CMM and LSM methods. Figure 1C clearly shows the effect of photon noise on lifetime precision, introducing errors that increase from ∼50 to ∼300 ps as photon counts are reduced from 5000 to 100. It shows, however, that no discernible bias is introduced by the CMM estimation across the signal range investigated.

In Figure 1D, the *F*‐value is plotted as a function of photon counts, for two levels of background noise and for both LSM and CMM. We observe that CMM determines the lifetime with constant photon efficiency across the whole range of counts. When background noise is present at Ap = 5% the *F*‐value increases from ∼1 to ∼1.23 from the case where no background noise is present. The LSM method, on the other hand, has lower photon efficiency which furthermore varies with photon counts. Figure S2 shows further data from these simulations, including lifetime images, accuracy and precision plots and data used to generate the *F*‐value graphs in Figure 1D.

The *F*‐value does not take into account the potential presence of bias (loss of accuracy) as highlighted by Li et al.^19^ We introduce an extension of the *F*‐value in a format that takes account of both precision and accuracy of the method:

(3) $$F^{\prime }=\sqrt{N}\frac{\sqrt{\sigma_{\tau }^{2}+\Delta_{\tau }^{2}}}{\tau }.$$

**FIGURE 2:**
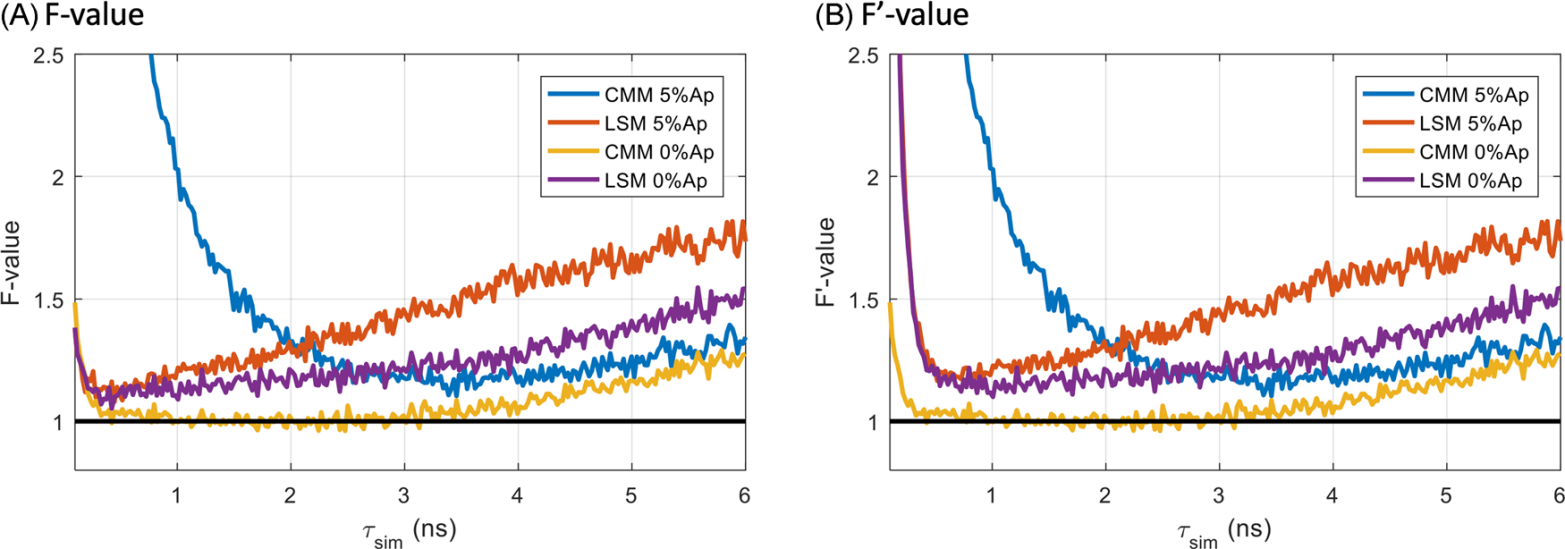
*F*‐value (A) and $F^{\prime }$‐value (B) as a function of the simulated lifetime ($\tau_{sim}$) for the CMM and LSM methods. The TCSPC decays were simulated with 5000 photons with 5% or 0% after‐pulsing background. When 5% after‐pulsing is applied, the background is corrected by removing the average of the first ∼20 times gates for CMM and by background level fitting for LSM. The *F* and $F^{\prime }$ values were estimated from 1024 repeats. The analysis window used the full acquisition window: 0–25 ns. Ap: after‐pulsing

Here, $\Delta_{\tau }$ is the difference between the mean of the measured and simulated lifetime. In combination, the *F*‐value and $F^{\prime }$‐value can be used as figures of merit in simulated data to investigate the overall performance of a lifetime estimation method. We then investigated the photon efficiency of the methods as a function of the lifetime extracted. Figure 2 shows plots of both *F* and $F^{\prime }$‐values as a function of the simulated lifetime for the CMM and LSM methods in the presence and absence of background noise, at 5% and 0% Ap, respectively. From Figure 2, we observe that the $F^{\prime }$‐value highlights a loss of accuracy of the LSM method at short lifetimes (<500 ps). We also note that, in absence of background, CMM performs close to optimally (F∼1) for lifetimes ranging from 0.5 to 4 ns and better than the LSM method. We note that LSM suffers more strongly from the presence of background than CMM in the lifetime range >2 ns. However, the opposite is true at shorter lifetimes (<2 ns), where the precision of the CMM lifetime estimation rapidly worsens.

Figure 2 shows, however, that CMM suffers from a loss of photon efficiency at short lifetimes (here <2.5 ns). The reason for this is that, for short lifetimes, the background noise in the tail of the fluorescence decay accumulates and becomes a dominating source of error in the lifetime estimation.

### 2.2 Improvement of CMM performance by adaptive windowing

**FIGURE 3:**
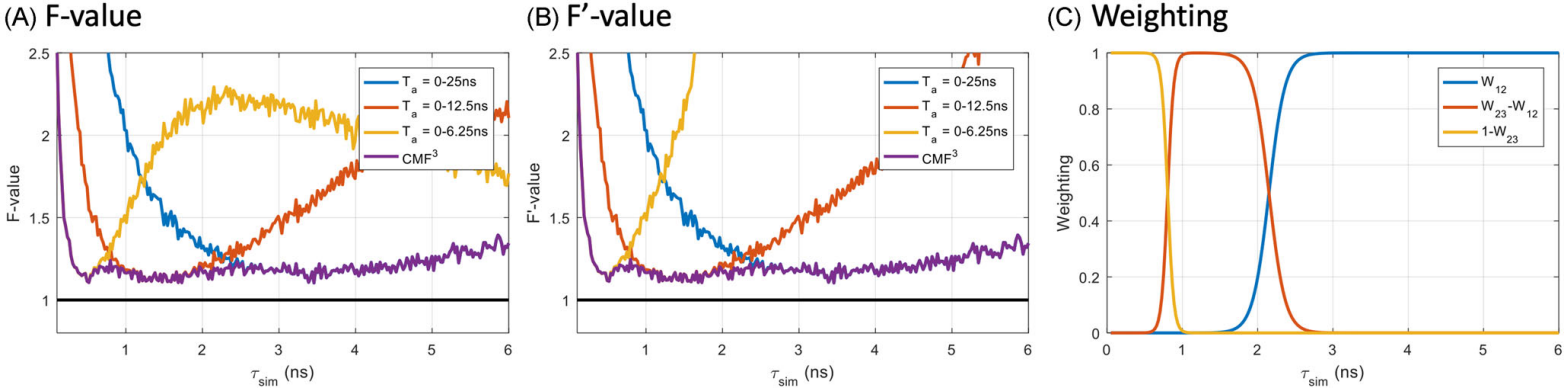
*F*‐value (A), $F^{\prime }$‐value (B) and weighting factors used for F3‐CMM (C) as a function of the simulated lifetime ($\tau_{sim}$). CMM was computed with three different analysis window sizes. The TCSPC decays were simulated with 5000 photons with 5% after‐pulsing background. The *F* and $F^{\prime }$ values were estimated from 1024 repeats. Ap: after‐pulsing

The loss of photon efficiency of CMM in the short lifetime range can be mitigated through the use of shorter analysis windows, reducing cumulative noise and thus improving the precision of the method for short lifetimes. In Figure 3A,B, we show the *F* and $F^{\prime }$‐values for CMM for three different analysis windows ($T_{a}$ = 0–25 ns, 0–12.5 ns and 0–6.25 ns).

We observe that each analysis window performs optimally only for a certain range of lifetimes. However, no single analysis window performs well over the complete range of lifetimes considered here. This then highlights a problem with CMM since within a single FLIM acquisition lifetimes can vary greatly from pixel to pixel. When using a wide analysis window, a large standard deviation is obtained for short lifetimes. However, when using a small analysis window an inaccurate (biased) lifetime is obtained for long lifetimes. Here, to address this problem, we introduce F3‐CMM, which combines the lifetime estimation from all three analysis windows and produces a composite lifetime image with improved precision and accuracy throughout the complete range of lifetime. The purple curve in Figure 3 was obtained from a weighted average of the results obtained from all three analysis window sizes following:

(4) $$\begin{array}{c} \begin{array}{cc} \tau_{F3-CMM}= & W_{12}\left(\tau \right)\tau_{CMM}^{T_{1}}+\left[W_{23}\left(\tau \right)-W_{12}\left(\tau \right)\right]\tau_{CMM}^{T_{2}}\\ & +\left[1-W_{23}\left(\tau \right)\right]\tau_{CMM}^{T_{3}}, \end{array} \end{array}$$

where *T*_1_, *T*_2_ and *T*_3_ correspond, respectively, to the analysis windows 25, 12.5 and 6.25 ns, $W_{ij}$ are the lifetime‐dependent weighting factors, computed from

(5) $$W_{ij}\left(\tau \right)=\frac{1}{1+e^{-b(\frac{\tau -\tau_{c}^{ij}}{\tau_{c}^{ij}})}},$$

where $\tau_{c}^{ij}$ is the lifetime cut‐off that separates analysis windows $T_{i}$ and $T_{j}$ and *b* is a ‘blending factor’ that determines the sharpness of the transition between adjacent weighting factors.

However, the weighting factors are lifetime‐dependent and therefore cannot be directly computed in real data from a sample of unknown lifetime. We find that the optimal weighting factors can be well estimated from the CMM (*T* = 12.5 ns) as it is sufficiently accurate and precise in the region of the cut‐off lifetimes (see Figure 3). Therefore, we use

(6) $$W_{ij}\left(\tau \right)\approx W_{ij}\left(\tau_{CMM}^{T_{2}}\right).$$

We note that an iterative method could be used to estimate the optimal weighting factors, instead of using the medium‐size window analysis but we do not expect this to lead to a significant improvement in the lifetime estimation.

**FIGURE 4:**
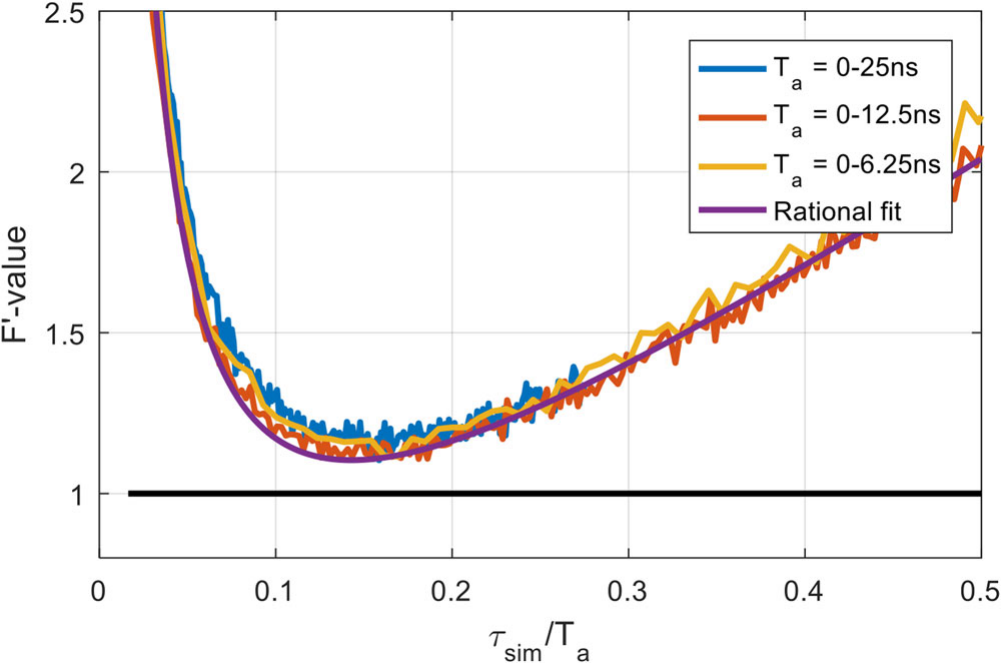
$F^{\prime }$‐value as a function of the ratio of the simulated lifetime and the analysis window with three analysis window sizes. The TCSPC decays were simulated with 5000 photons with 5% after‐pulsing background. The *F* and $F^{\prime }$ values were estimated from 1024 repeats. The rational fit is of the form: $F^{\prime }\left(\frac{\tau_{sim}}{T_{a}}\right)=A\frac{\tau_{sim}}{T_{a}}+B+C\frac{T_{a}}{\tau_{sim}}$, $R^{2}=0.9983$

In order to determine the lifetime range appropriate for a given analysis window size (and therefore the lifetime cut‐off $\tau_{c}^{ij}$), we plot the $F^{\prime }$‐value as a function of the ratio of the lifetime and the analysis window size as shown in Figure 4. This leads to a plot, the shape of which is invariant with analysis window size and which is well described by a rational function of the type

(7) $$F^{\prime }\left(\alpha \right)=A\alpha +B+\frac{C}{\alpha }.$$

**FIGURE 5:**
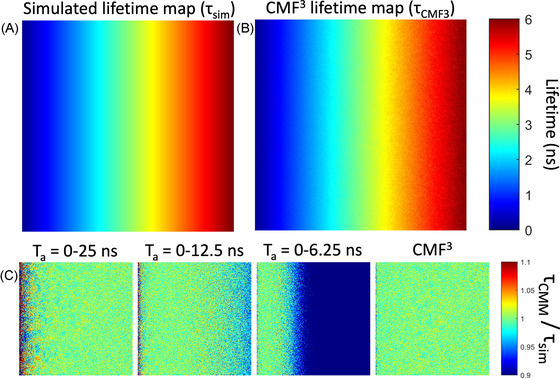
Performance analysis of F3‐CMM for simulated data and comparison with standard CMM. (A) Simulated lifetime map (representing ground truth). (B) Lifetime map extracted by F3‐CMM. (C) Comparison of CMM and F3‐CMM results with ground truth, expressed as the recovered lifetime divided by the simulated lifetime. The first three panels show CMM data for different analysis windows. The fourth panel corresponds to F3‐CMM. The TCSPC image data were simulated with 5000 photons with 5% after‐pulsing background

This analytical description may be used to determine lifetime cut‐offs $\tau_{c}^{ij}$ between two analyses windows ij, defined as the lifetime where the two curves cross and therefore

(8) $$\tau_{c}^{ij}=\sqrt{T_{a}^{i}T_{a}^{j}\frac{C}{A}},$$

where $T_{a}^{i}$ represents the effective analysis window size. The parameters *C* and *A* are relatively insensitive to the amount of Ap (see Figure S3) and can therefore be estimated from the $F^{\prime }$‐value plot as a function of lifetime. For all results presented here, we used: *A* = 3.218 and *C* = 0.07339. We first tested the performance of our method in silico, by simulating TCSPC image data with a lifetime gradient. These simulated data were subsequently analysed with F3‐CMM and CMM. The results are shown in Figure 5. As expected, each of the three analysis window performs well (low noise and low bias) only in specific regions of the lifetime map. The large window ($T_{a}$ = 0–25 ns) shows large noise at low lifetimes (0–1 ns range) and the small analysis window ($T_{a}$ = 0–6.25 ns) has large bias towards shorter lifetime in the long lifetime region (>2 ns). F3‐CMM, on the other hand, is able to estimate the lifetime correctly over the entire lifetime range.

We also generated in silico data sets to simulate challenging acquisition conditions. In Figure S4, we present a data set containing three different lifetime values (0.5, 1.5 and 3.5 ns) and variable signal photon numbers (∼20, ∼50 and ∼120, respectively, corresponding to ∼5 photons in the maximum bin in all decays) with a 5% Ap background. It is clear that F3‐CMM offers improved performance over CMM and LSM analyses in terms of precision and accuracy.

We also wanted to characterize the method's performance on the cases where observed decays are incomplete, for instance when measuring long lifetimes with high repetition rate lasers. Figure S5 shows simulations of both complete and incomplete decays. In the case of the complete decay, we simulated a lifetime of 0.5 ns at a laser repetition rate of 20 MHz. For the incomplete decay, we simulated a lifetime of 5 ns at a laser repetition rate of 80 MHz. Both cases were generated in the presence of background in the form of 5% Ap, with a varying levels of photon counts from 100 to 5000 photons, as we did before. We observe that the number of photons had minimal impact on the *F*‐number in the range we considered and that F3‐CMM accurately predicted the lifetimes for complete decays, with minimal biases even at high background. On the other hand, incomplete decays showed a bias towards shorter lifetimes and a high *F*‐value, as is commonly known for other CMM approaches. For analysing incomplete decays, fitting an incomplete decay model may be a more accurate approach here.

### 2.3 Validation of the approach on experimental data

**FIGURE 6:**
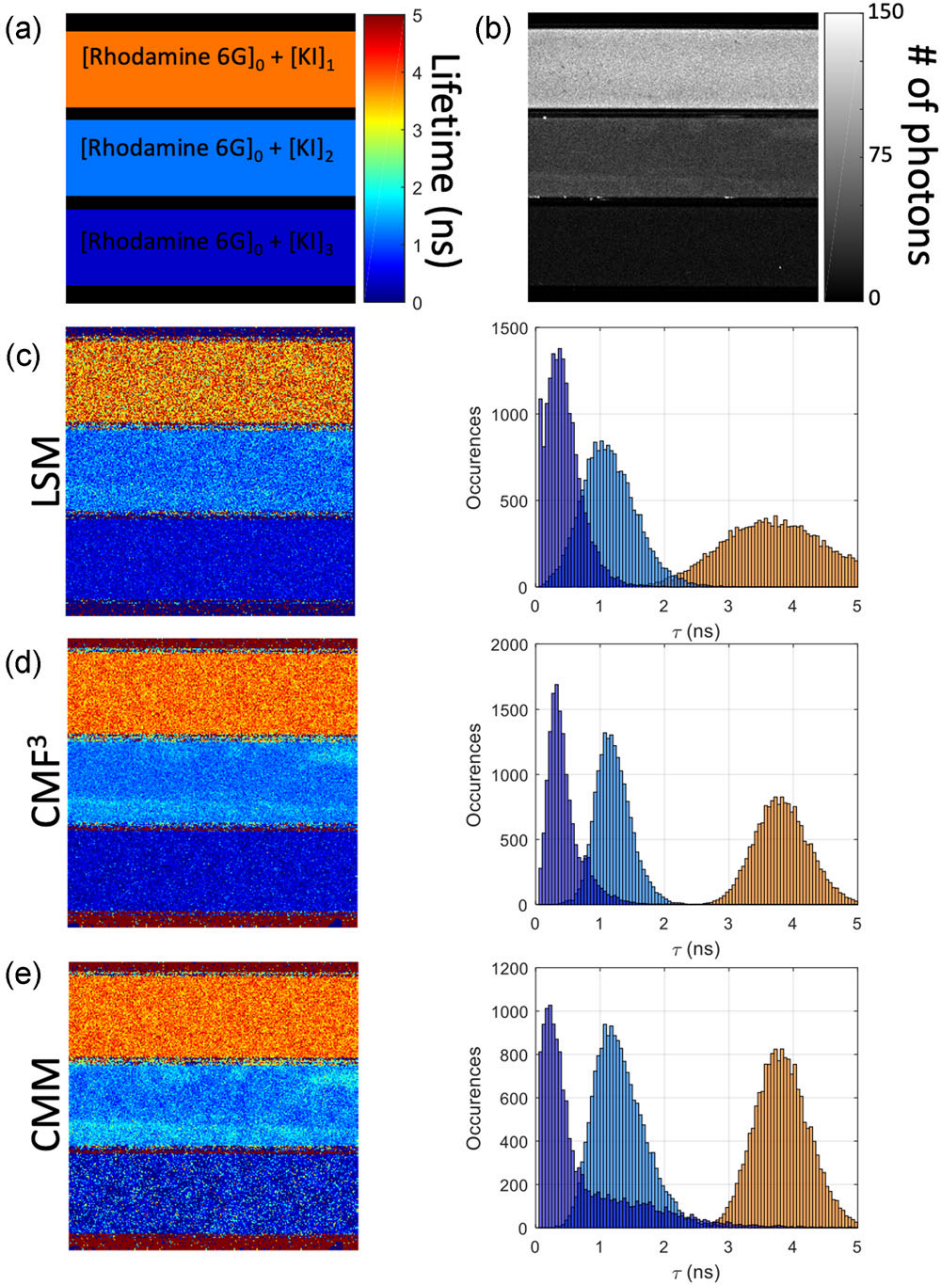
Comparison of F3‐CMM, LSM and conventional CMM on Rhodamine 6G data. (A) Diagram representing the set‐up of the capillary sample. Three rectangular capillaries filled with Rhodamine 6G and increasing concentrations of quencher KI were used. (B) Total intensity image obtained from the TCSPC data set. (C–E) Recovered lifetime images (left) and corresponding lifetime histograms (right). The histograms correspond to a strip of 67 pixels wide centred on each capillary. The lifetime scale is indicated as in (A). The background in the images represented an after‐pulsing in the range of 1–4%

Next, we validated the method on experimental data. For this purpose, we used lifetime calibration solutions based on Rhodamine 6G dye solutions containing varying concentrations of potassium iodide (KI) as a fluorescence quencher.^25^ The calibration solutions obtained this way have been shown to provide a large range of lifetimes and allow titration of the quencher.^6^, ^26^ Here, we filled three transparent glass capillaries with varying mixtures of Rhodamine 6G and KI and imaged them side‐by‐side in a single field‐of‐view using our custom‐built TCSPC confocal microscope.^27^ The results are shown in Figure 6. The recovered lifetime images and the histograms demonstrate the superior performance of the F3‐CMM approach, leading to sharper lifetime distributions and less noisy images compared to LSM and standard CMM. Conventional CMM leads to a broader distribution at short lifetimes (∼0.3 ns), where there is also bias, evident from the large tail extending to lifetimes beyond 3 ns (see Figure 6E). This tail is absent in the F3‐CMM approach.

The means and standard deviations for the recovered lifetimes are compared in Table 1 for the different methods. Here again, the F3‐CMM lifetime fraction map shows the best recovery of lifetime across the whole range of lifetimes. The lifetime values obtained for the conventional CMM, LSM and F3‐CMM are in good agreement with each other (∼ 3.8, ∼1.2 and ∼0.3 ns, respectively, for capillary ${\left[KI\right]}_{1}$, ${\left[KI\right]}_{2}$ and ${\left[KI\right]}_{3}$), but the standard deviations varied significantly. For the long lifetime, the standard deviation was halved by the CMM approaches compared to LSM. Also, CMM and F3‐CMM performed identically, as expected since conventional CMM uses the full analysis window ideal for long lifetimes. For mid‐range lifetime (∼1.2 ns), the F3‐CMM shows an improvement of 1.43‐fold on the standard deviation over CMM. Finally, for the short lifetime (∼0.3 ns), F3‐CMM leads to a twofold improvement of the standard deviation over CMM. The estimated *F*‐value shown in Table 1 quantitatively highlights the photon efficiency of F3‐CMM.

**TABLE 1:** Means and standard deviations of the lifetime estimated for each capillary for all three methods

	[KI]	LSM	F3‐CMM	CMM
$\bar{\tau }\pm \sigma_{\tau }$	${\left[KI\right]}_{1}=0$ mM	3.70 ± 1.20	3.81 ± 0.59	3.81 ± 0.59
$\bar{\tau }\pm \sigma_{\tau }$	${\left[KI\right]}_{2}=39$ mM	1.10 ± 0.58	1.18 ± 0.37	1.24 ± 0.53
$\bar{\tau }\pm \sigma_{\tau }$	${\left[KI\right]}_{3}=200$ mM	0.33 ± 0.43	0.34 ± 0.24	0.12 ± 0.51
*F*‐value	${\left[KI\right]}_{1}=0$ mM	3.32	1.59	1.59
*F*‐value	${\left[KI\right]}_{2}=39$ mM	3.12	1.84	2.52
*F*‐value	${\left[KI\right]}_{3}=200$ mM	4.23	2.35	13.97

*Note*: The mean lifetime ($\bar{\tau }$) and its standard deviation ($\sigma_{\tau }$) were estimated by fitting a Gaussian function to the lifetime distribution. The *F*‐value can then be estimated in each case by using the average number of photons in the decays from each capillary (105, 35 and 11, respectively, for ${\left[KI\right]}_{1}$, ${\left[KI\right]}_{2}$ and ${\left[KI\right]}_{3}$).

We also compared the performance of F3‐CMM against LSM method for a set of diffraction‐limited images of *Convallaria* *majalis* in Figure S6 and observed equivalent performance in terms of measured average lifetimes.

## 3 CONCLUSIONS

Our simulations and experimental data clearly show the potential of CMM and, in particular, of the F3‐CMM extension as a method to accurately and precisely estimate the fluorescence lifetime from single exponential decays over a wide lifetime dynamic range. The photon efficiency (indicated by the $F^{\prime }$‐value) is higher than for LSM for lifetime ranges typically encountered in biological fluorescence experiments, improving precision and accuracy of the lifetime determination for the same photon budget. Generally, the CMM algorithm is also computationally efficient and faster than state‐of‐the‐art LSM implementations and has been implemented ‘on‐chip’ with FLIM instrumentation.^19^, ^20^, ^28^

The disadvantage of the CMM approach is that multi‐exponential decays cannot be distinguished and CMM will provide an estimate of an average lifetime instead. If this is desired, a global analysis and multi‐exponential fitting with LSM are powerful alternatives. However, the average lifetime is often sufficiently informative to reveal functional information and, with appropriate calibration, is quantitative for the measurement of absolute ligand concentrations.^1^ F3‐CMM is robust with respect to changes in background noise but the parameters used to compute the weighting factors can be adjusted if exceptionally large amounts of background noise are present. F3‐CMM will be especially beneficial when background noise is present in the data set, for example when using SPAD arrays, which typically feature dark count rates that are typically higher than that encountered with photomultiplier tubes or with hybrid detectors.^22^ In conclusion, we have shown that using an adapted analysis window with CMM lifetime estimation leads to excellent photon efficiency with high precision and accuracy over a large range of lifetimes. We have introduced the $F^{\prime }$‐value as a measure of overall photon efficiency that takes into account both the precision and the accuracy of the method. It also helps identifying the range of lifetimes that the method can extract with high fidelity. In addition to its high photon efficiency, the speed afforded by F3‐CMM offers potential for quantitative dynamic, live cell measurement applications and for on‐chip, video‐rate implementation of FLIM analysis.

## 4 MATERIALS AND METHODS

### 4.1 Monte‐Carlo simulation of TCSPC data set

TCSPC data were generated using a Monte‐Carlo simulations in MATLAB from the probability density functions (PDF) of the photon excitation and emission times, which were represented by the instrument response function (IRF) model and the fluorescence decay model, respectively. The cumulative probability densities (CPDF) were calculated from the probability densities as shown in Figure S1. For the photon emission time, we used

(9) $$PDF_{em}\left(t\right)=\frac{1}{\tau }e^{-\frac{t}{\tau }}$$

and

(10) $$CPDF_{em}\left(t\right)=1-e^{-\frac{t}{\tau }}.$$

For the Gaussian IRF, we used

(11) $$PDF_{ex}\left(t\right)=\frac{1}{\sigma_{IRF}\sqrt{\pi }}e^{-\frac{{\left(t-t_{0}\right)}^{2}}{\sigma_{IRF}^{2}}}$$

and

(12) $$CPDF_{ex}\left(t\right)=\frac{1}{2}\left[erf\left(\frac{t_{0}}{\sigma_{IRF}}\right)-erf\left(\frac{t_{0}-t}{\sigma_{IRF}}\right)\right],$$

where ex refers to excitation, em refers to emission and erf refers to the error function. Two numbers $x_{1}\left(i\right)$ and $x_{2}\left(i\right)$ were generated by a pseudo‐random number generator (PRNG) from a uniform distribution between 0 and 1 using the MATLAB function rand. These numbers were used to obtain the corresponding emission and excitation times, $t_{em}$ and $t_{ex}$, respectively, as shown in Figure S1. The photon arrival time is the sum of the two: $t_{arr}=t_{em}+t_{ex}$. The number of photons in the background $N_{bkg}$ was determined from the after‐pulsing (Ap) and the total number of photons (*N*)

(13) $$N_{bkg}=Ap*N.$$

The background noise level was simulated by randomly distributing (uniformly distributed across the acquisition window) the background photon arrival times across the acquisition window. The $N+N_{bkg}$ arrival times were binned to form a histogram of arrival times. Then, the arrival times beyond the acquisition window were wrapped around applying a modulo operation using the temporal period between laser pulses. The IRF was simulated by setting the emission time ($t_{em}$) to zero. The simulation tool can generate a range of lifetimes and a range of photon numbers in an image of any size and then save it as 16‐bit TIFF stacks using the OMERO MATLAB utilities. The simulation tool depends on the bioformats package, which is free, open‐source, and easy to install from https://docs.openmicroscopy.org/bio‐formats/6.1.0/users/matlab/index.html. The TIFF stack can then be used for CMM analysis or imported into FLIMfit^8^ for LSM analysis. The code is available on the author's GitHub: https://github.com/Romain‐Laine/TCSPC‐image‐simulation.

### 4.2 Computation of the CMM and F3‐CMM algorithms

Prior to computing the centre‐of‐mass, the backgrounds were removed from each decay by calculating the average of the first few time bins before the rising edge of the decay and subtracting it from the decay. Then, the lifetime extracted by CMM was estimated by calculating the centre‐of‐mass lifetime estimator, $\tau_{CM}$, of the corresponding decay with the chosen analysis window.

(14) $$\tau_{CM}=\frac{\sum_{bin=0}^{n-1}I\left(t_{bin}\right)\Delta bin}{\sum_{bin=0}^{n-1}I\left(t_{bin}\right)}+\frac{\Delta bin}{2},$$

where $I(t_{bin})$ is the fluorescence intensity measurement in the temporal bin and Δbin is the bin size. The centre‐of‐mass of the IRF decay $\tau_{IRF}$ was also computed in order to remove the effect of the IRF on the lifetime estimation. The CMM lifetime $\tau_{CMM}$ was then obtained by subtracting the IRF lifetime.

(15) $$\tau_{CMM}=\tau_{CM}-\tau_{IRF.}$$

Then the lifetime corrected for the finite analysis window, $\tau_{CMM}^{corr}$, was computed by iterative method as follows:

(16) $$\tau_{k+1}=\tau_{CMM}+T_{a}\frac{e^{-\frac{T_{a}}{\tau_{k}}}}{1-e^{-\frac{T_{a}}{\tau_{k}}}},$$

where *k* is the number of iterations, $\tau_{k}$ is the lifetime at the *k*th iteration, $T_{a}$ is the effective analysis window size and with $\tau_{0}=\tau_{CMM}$. The effective analysis window is given by the difference between the total analysis window and $\tau_{IRF}$. In practice, we noticed that 10 iterations were sufficient to obtain unbiased estimates of the lifetime within the range of lifetimes considered here, therefore $\tau_{CMM}^{corr}=\tau_{10}$. The F3‐CMM is computed as described in the main text. A blending factor of *b* = 20 was found to work well in most cases by providing a sharp transition between the lifetime images obtained from different analysis windows. The analysis script was written as an open‐source Fiji^30^ macro tool with the aim to integrate FLIM analysis with other versatile microscopy image analysis and make it generally more accessible to end users, in a similar spirit to FLIMJ.^29^ The analysis tool is distributed on the GitHub page of the author: https://github.com/Romain‐Laine/F3‐CMM‐FLIM‐analysis.

### 4.3 FLIM imaging of Rhodamine 6G and *Convallaria* samples

Rhodamine 6G (Sigma, R4127) were prepared in increasing concentrations of KI (Sigma‐Aldrich, 60400) in accordance to Hanley et al.^25^ The dye and quencher mixture were freshly prepared and then used to fill up hollow rectangle capillaries (CM Scientific, ID 0.10 × 1.00 mm). The capillaries were placed side by side on the microscope stage and imaged on a custom‐made TCSPC microscope as previously described.^27^ An Olympus 2× objective with 0.08 NA was used. FLIM images were acquired over a 140‐s integration time. For imaging of slide‐mounted *Convallaria majalis* samples, an Olympus 60× oil immersion objective with 1.40 NA was used. The laser source was a Fianium SC400‐4 set to a 20‐MHz repetition rate. A 510‐nm excitation wavelength and 560‐nm emission wavelength (Semrock 560/25 filter) were used for both experiments.

## Supporting information

Figure S1: TCSPC data simulation. PDF: probability density function, CPDF: cumulative probability density function, ex: excitation, em: emission, erf: error function, PRNG: Pseudo‐random number generator, N: total number of photons generated for the decay. x1 (i) and x2(i) are the pseudo‐random numbers generated for the calculation of the arrival time of the photon i
Figure S2: Lifetime images, accuracy and precision plots for CMM and LSM as a function of photon counts with and without background noise (5% Ap and 0% Ap respectively).
Figure S3: F'‐value as a function of the simulated lifetime in terms of fraction of the analysis window
Figure S4: Comparison of CMM, F3‐CMM with LSM on in‐silico data.
Figure S5: Comparison of F3‐CMM performance on in silico data with complete and incomplete decays.
Figure S6: Comparison of F3‐CMM and LSM performance on diffraction‐limited images of Convallaria majalis, a standard microscopy reference sample featuring a complex mixture of fluorescence decays.
