# Supplementary material for: A method for the fast and photon‐efficient analysis of time‐domain fluorescence lifetime image data over large dynamic ranges
Source: J Microsc. 2022 Jun 23;287(3):138–47. doi: 10.1111/jmi.13128 (PMC9544871; doi:10.1111/jmi.13128)
Supplement: Supplementary file 1 — Figure S1: TCSPC data simulation. PDF: probability density function, CPDF: cumulative probability density function, ex: excitation, em: emission, erf: error function, PRNG: Pseudo‐random number generator, N: total number of photons generated for the decay. x1 (i) and x2(i) are the pseudo‐random numbers generated for the calculation of the arrival time of the photon i Figure S2: Lifetime images, accuracy and precision plots for CMM and LSM as a function of photon counts with and without background noise (5% Ap and 0% Ap respectively). Figure S3: F'‐value as a function of the simulated lifetime in terms of fraction of the analysis window Figure S4: Comparison of CMM, F3‐CMM with LSM on in‐silico data. Figure S5: Comparison of F3‐CMM performance on in silico data with complete and incomplete decays. Figure S6: Comparison of F3‐CMM and LSM performance on diffraction‐limited images of Convallaria majalis, a standard microscopy reference sample featuring a complex mixture of fluorescence decays. [file JMI-287-138-s001.pdf]

A method for the fast and photon-efficient analysis of time domain fluorescence lifetime image data over large dynamic ranges

Romain F. Laine[1,2,*], Chetan Poudel[1,3,*] and Clemens F. Kaminski[1]

[1]Laser Analytics Group, Department of Chemical Engineering and Biotechnology, University of Cambridge, Philippa Fawcett Drive, Cambridge, CB3 0AS, UK

[2]Current address: Medical Research Council Laboratory for Molecular Cell Biology (LMCB), University College London, Gower Street, London, WC1E 6BT, UK

[3]Current address: Department of Chemistry, University of Washington, Seattle, WA, 98195, USA

*These authors contributed equally to this manuscript.

Supplementary information

Figure S1: Description of the TCSPC simulation.

Figure S2: Lifetime images, accuracy and precision plots for CMM and LSM as a function of photon counts.

Figure S3: F'-value as a function of the simulated lifetime in terms of fraction of the analysis window.

Figure S4: Comparison of CMM, F3-CMM with LSM on *in silico* data.

Figure S5: Comparison of F3-CMM performance on in silico data with complete and incomplete decays.

Figure S6: Comparison of F3-CMM and LSM performance on diffraction-limited images of *Convallaria majalis*.

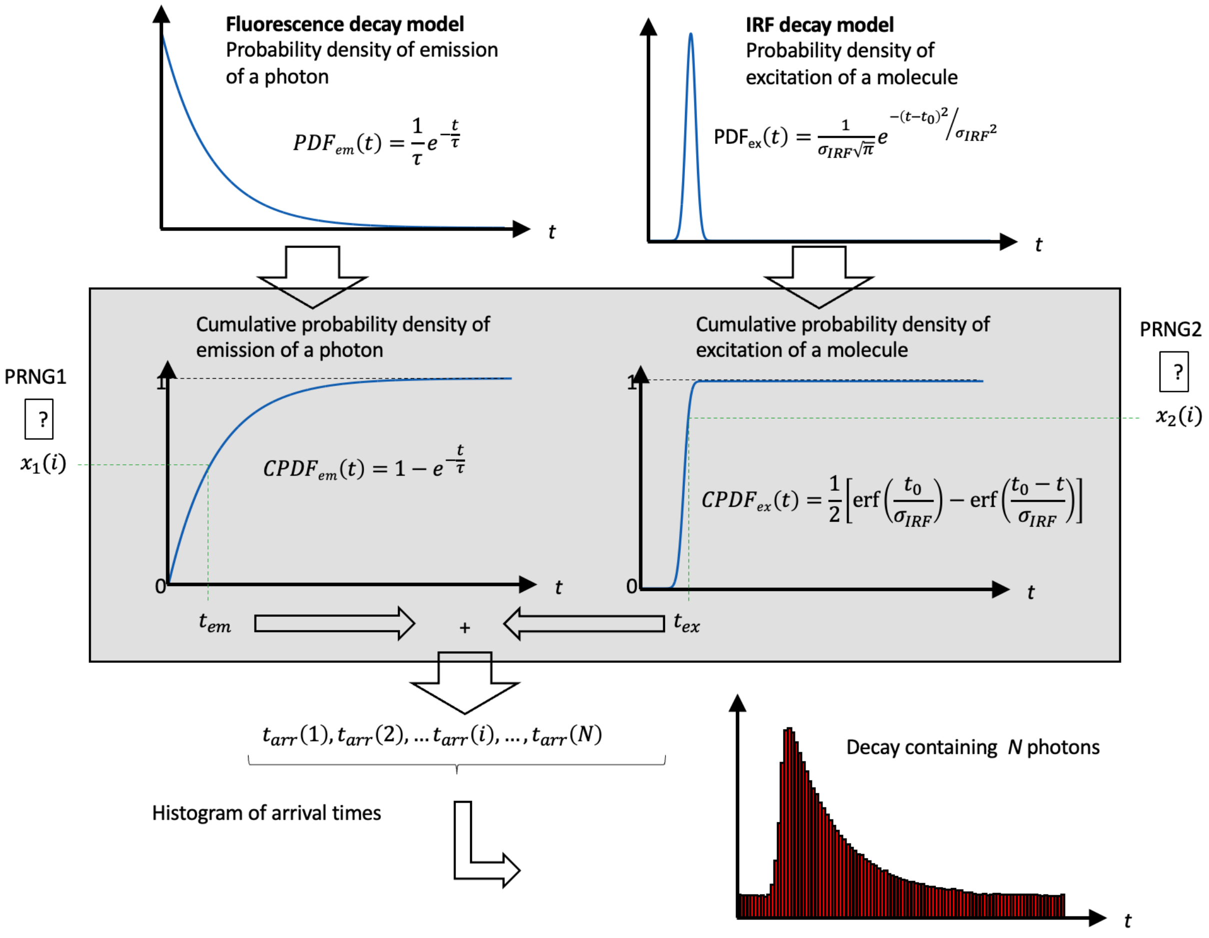

Figure S1: TCSPC data simulation. PDF: probability density function, CPDF: cumulative probability density function, ex: excitation, em: emission, erf: error function, PRNG: Pseudo-random number generator, N: total number of photons generated for the decay. x_1(i) and x_2(i) are the pseudo-random numbers generated for the calculation of the arrival time of the photon i.

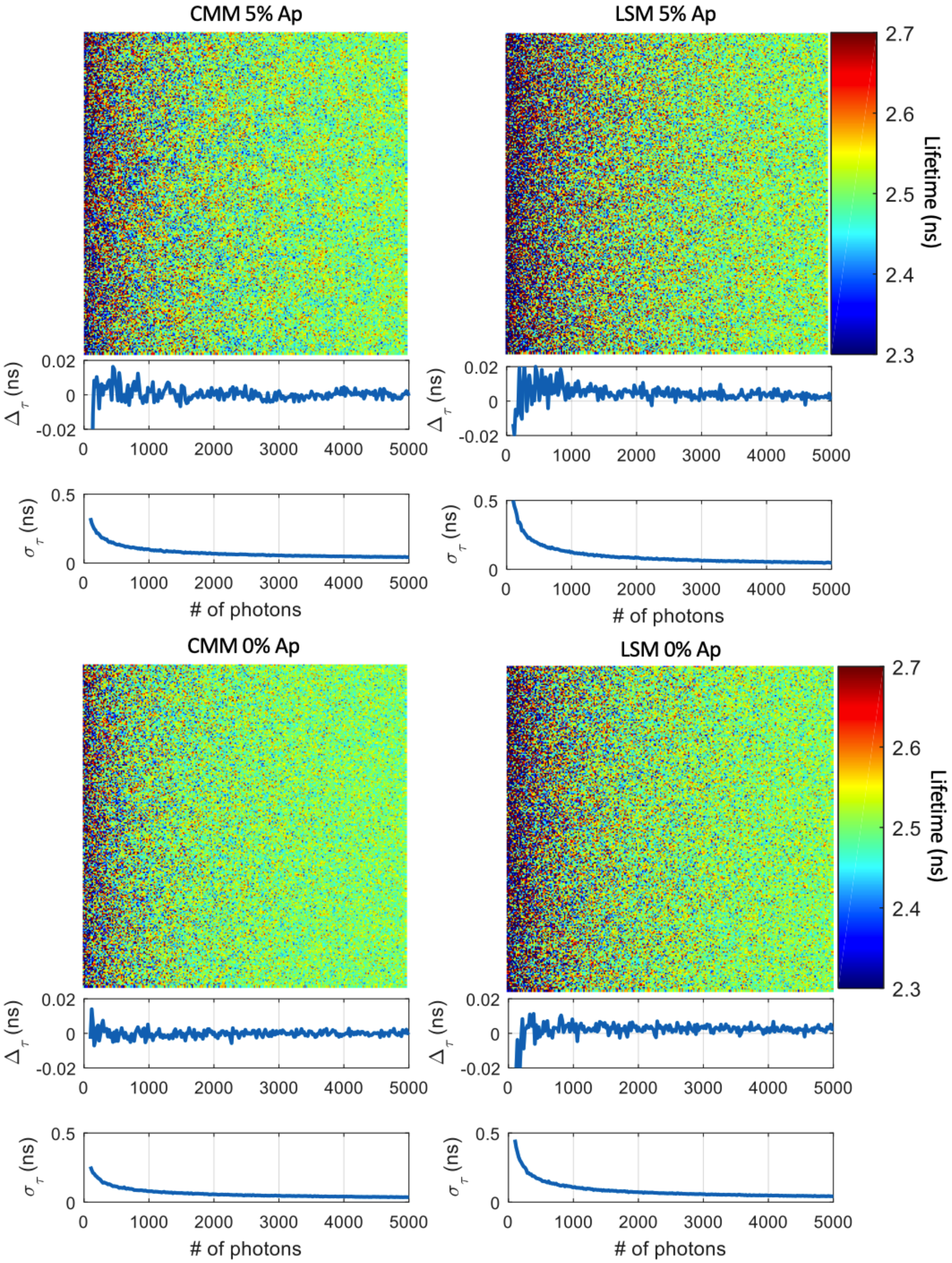

Figure S2: Lifetime images, accuracy and precision plots for CMM and LSM as a function of photon counts with and without background noise (5% Ap and 0% Ap respectively).

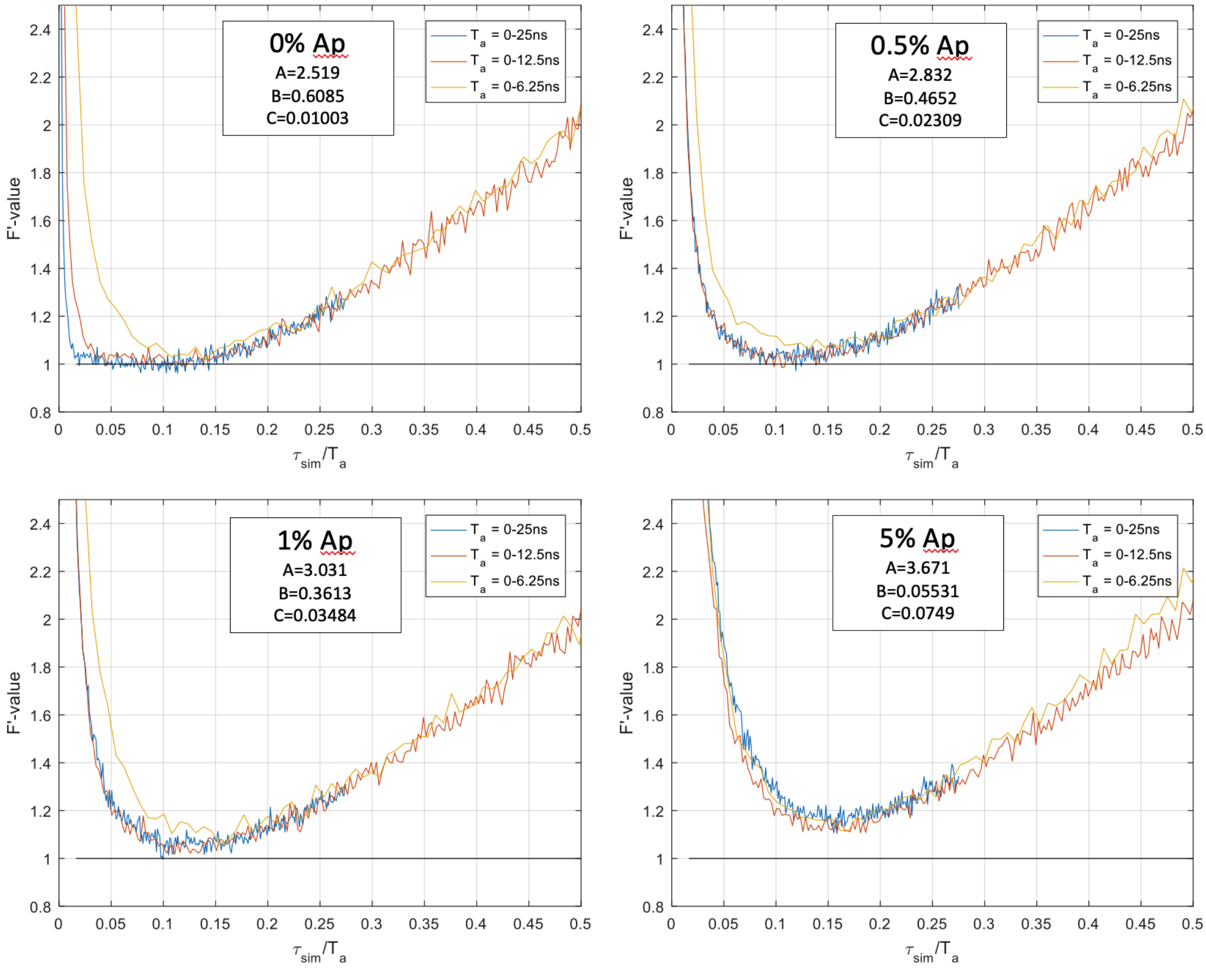

Figure S3: F'-value as a function of the simulated lifetime in terms of fraction of the analysis window. Four different levels of noise are investigated (0%, 0.5%, 1% and 5% of after-pulsing). N = 5,000 photons. The parameters of the rational function describing the curves are shown in the inset.

$$F'\left(\frac{\tau_{sim}}{T_a}\right) = A\frac{\tau_{sim}}{T_a} + B + C\frac{T_a}{\tau_{sim}}$$

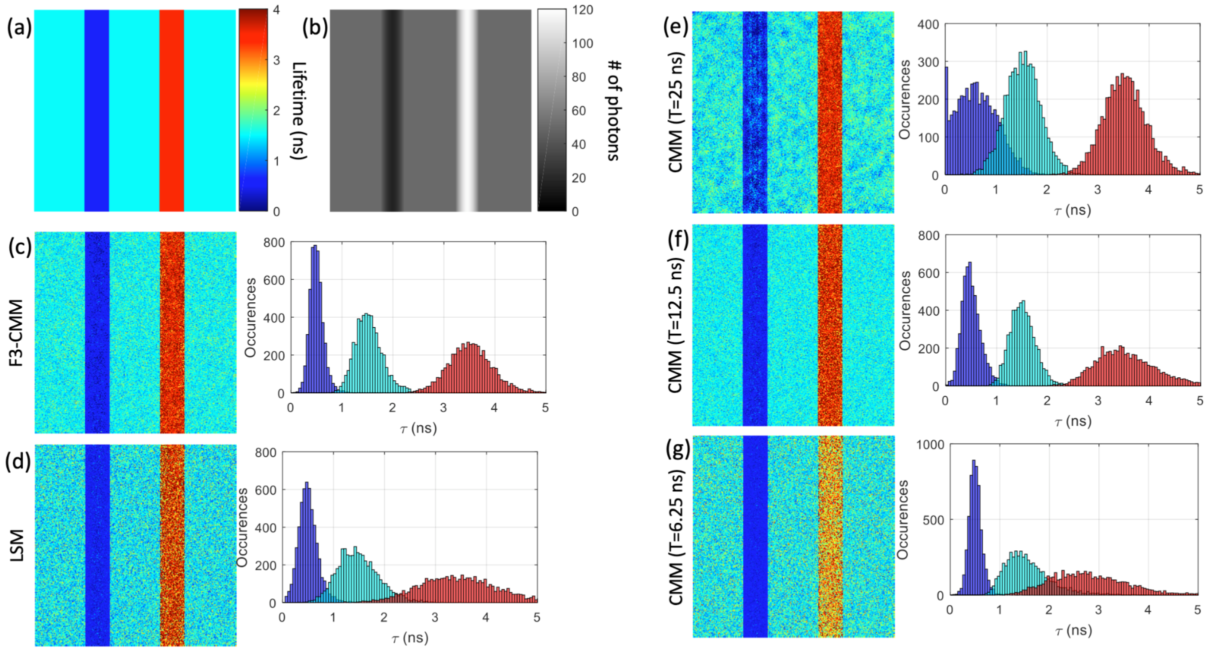

Figure S4: Comparison of CMM, F3-CMM with LSM on *in-silico* data. The lifetimes were 1.5 ns, 0.5 ns and 3.5 ns. The number of photons were adjusted such that the shorter lifetimes had fewer photons, equal to a similar number of photons in the maximum bins for all lifetimes (~5 photons). The histograms of lifetimes shown on the right of each FLIM image correspond to a strip of 20 pixels wide centered on the 0.5 ns strip (dark blue histogram), centered on the 1.5 ns strip (light blue histogram), and centered on the 3.5 ns strip (red histogram).

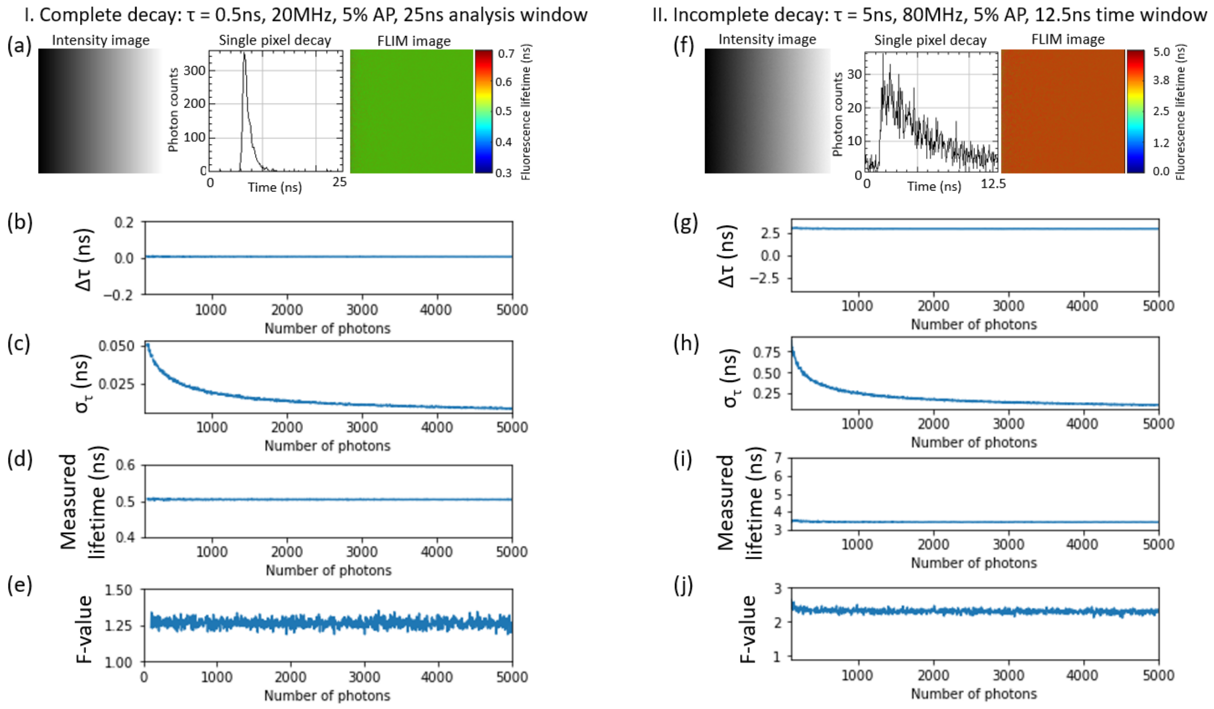

Figure S5: Comparison of F3-CMM performance on *in silico* data with complete and incomplete decays. In the case of the complete decay (I), we simulated a lifetime of 0.5ns at a laser repetition rate of 20MHz. For the incomplete decay (II), we simulated a lifetime of 5ns at a laser repetition rate of 80MHz. (a,e) The number of photons increases from 100 to 5000 photons from left to right in the intensity images. This allows for comparing the performance of F3-CMM in a large range of photon counts. The fluorescence decay from a single pixel at the center of the image is shown as an example. (b,f) Deviation ($\Delta\tau$) of measured lifetime from the simulated lifetime as a function of the number of photons (from 1,024 repeats). (c,g) Standard deviation ($\sigma\tau$) of the measured lifetime as a function of the number of photons (from 1,024 repeats). (d,h) The measured lifetime as a function of the number of photons (from 1,024 repeats). (e,j) F-value as a function of the number of photons (from 1,024 repeats) in the presence of background (5% Ap).

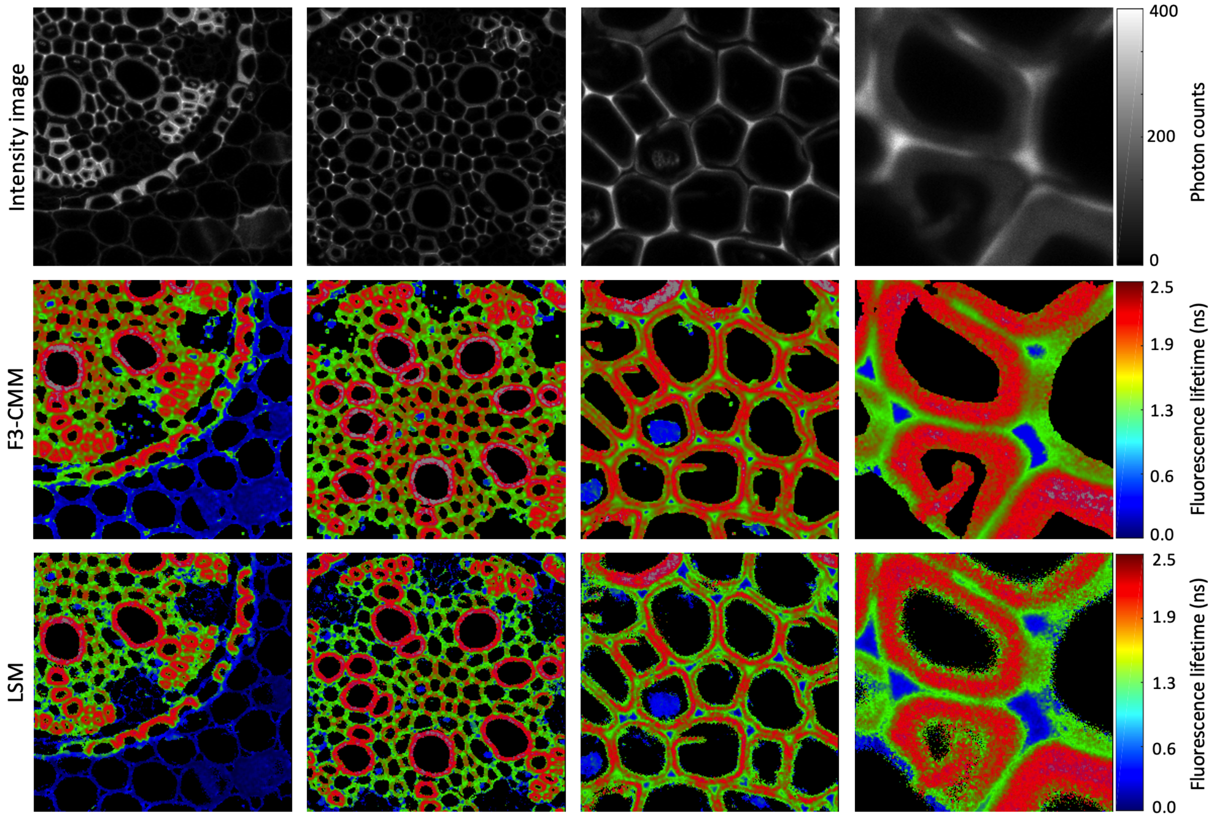

Figure S6: Comparison of F3-CMM and LSM performance on diffraction-limited images of *Convallaria majalis*, a standard microscopy reference sample featuring a complex mixture of fluorescence decays. Four images were acquired at different zoom factors (top row). The performance of F3-CMM (middle row) in analyzing the average lifetime of a complex mixture is visually/qualitatively very similar to LSM analysis (bottom row) performed using the open-source FLIMfit package.
